# Supplementary material for: Distinguishing preferences of human APOBEC3A and APOBEC3B for cytosines in hairpin loops, and reflection of these preferences in APOBEC-signature cancer genome mutations
Source: Nat Commun. 2024 Mar 18;15:2369. doi: 10.1038/s41467-024-46231-w (PMC10948833; doi:10.1038/s41467-024-46231-w)
Supplement: Supplementary file 1 — Supplementary Information [file 41467_2024_46231_MOESM1_ESM.pdf]

A Purification of A3B-CTD

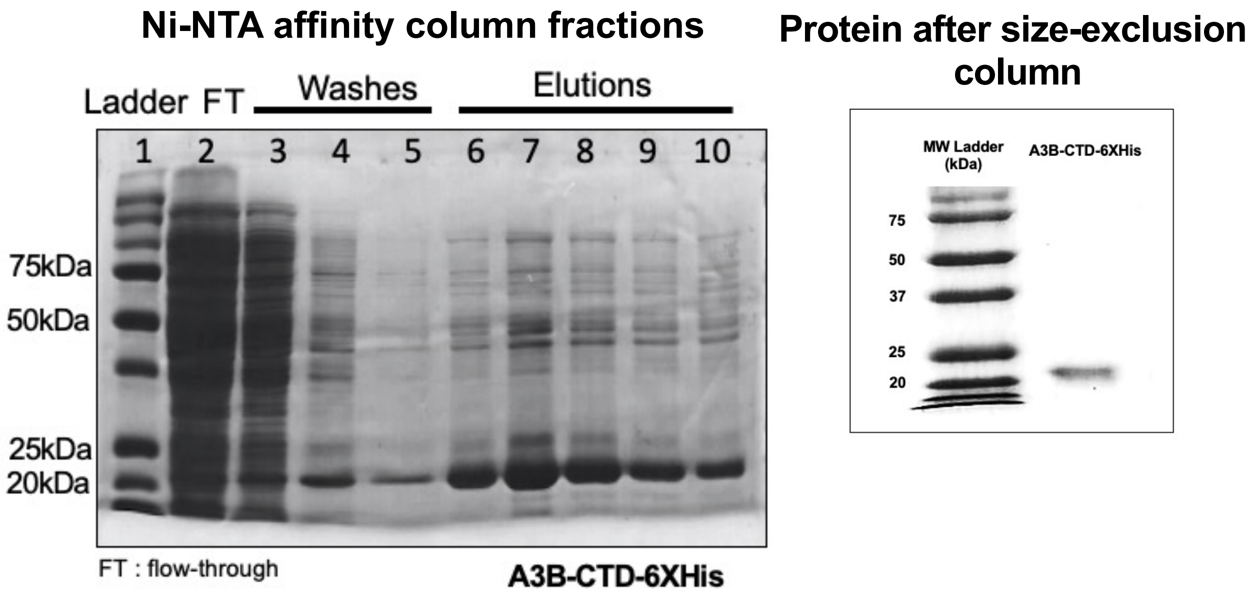

B Purification of A3A

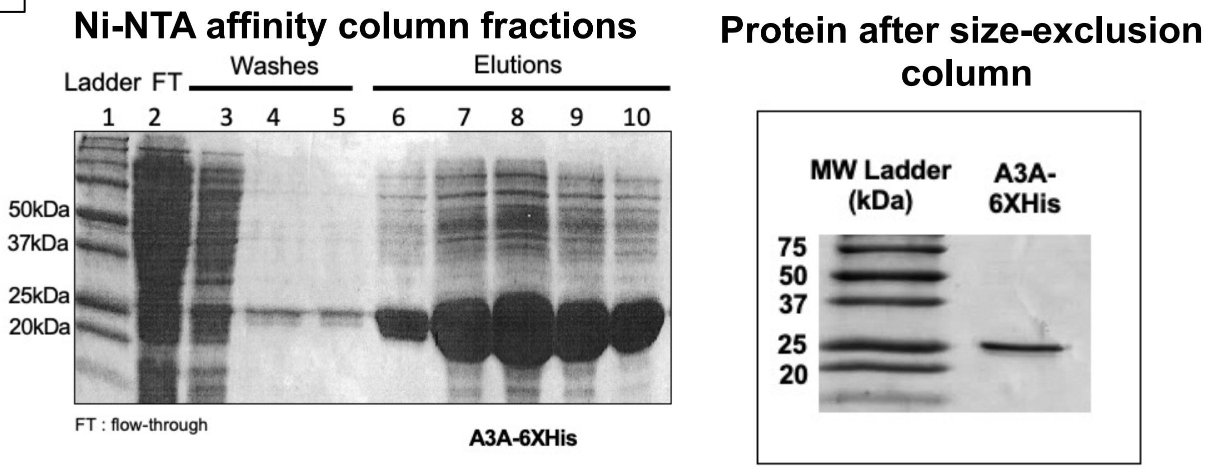

Supplementary Fig. S1

Purification of A3A and A3B-CTD proteins. Poly-His tagged A3A and A3B-CTD proteins were purified to apparent homogeneity using Ni-NTA affinity chromatography followed by separation on a size-exclusion column.

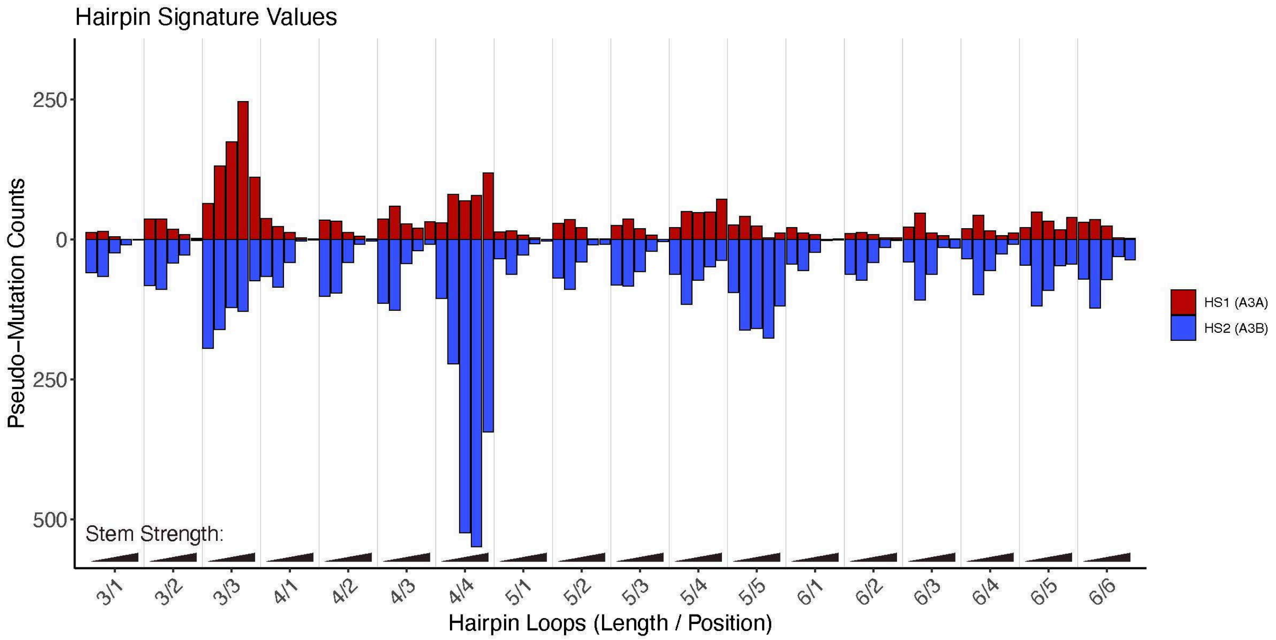

Supplementary Fig. S2

Hairpin Signature Values for A3A and A3B from UPD-seq. The sum of pseudo-mutation* counts in four A3A samples and six A3B-Full samples. These sets of counts help define hairpin signature 1 (HS1; A3A) and hairpin signature 2 (HS2; A3B). Vertical lines separate the values into 18 groups based on hairpin loop length and position. For example, 3/1 has cytosine at 5' end of a trinucleotide loop. Within each group, the values are ordered by increasing stem strength. The bins for stem strengths are: 1-4, 5-7, 8-11, 12-15, and 16+.

*Pseudo-mutations are positions with uracilation fraction ≥0.03

A

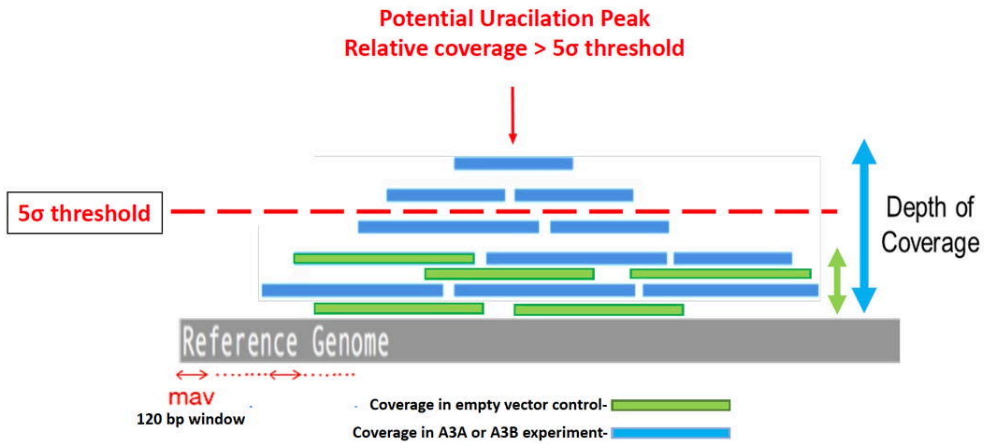

B

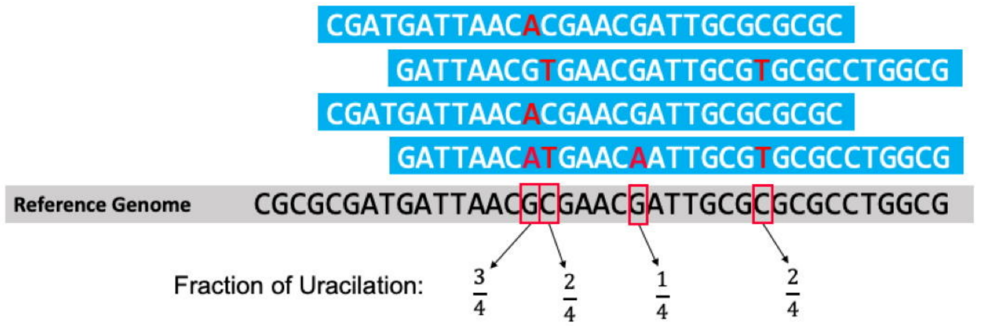

Supplementary Fig. S3

Graphical explanation of uracilation peaks and uracilation index. A. A uracilation peak is detected by comparing the depth of coverage in UPD-seq experiment with that of empty vector control. The NDC2 algorithm calculates the difference in the depth of coverage across the genome using a 120 base pair moving window. “mav” is moving average. A uracilation peak is defined as a region in the genome where relative coverage of the experimental sample is five standard deviations above average. **B.** At any C:G pair in the genome the fraction of sequencing reads that have an T:A pair is calculated. This number, when multiplied with 1,000 gives uracilation index, UI.

Overlap of Uracilation Peaks with TSS

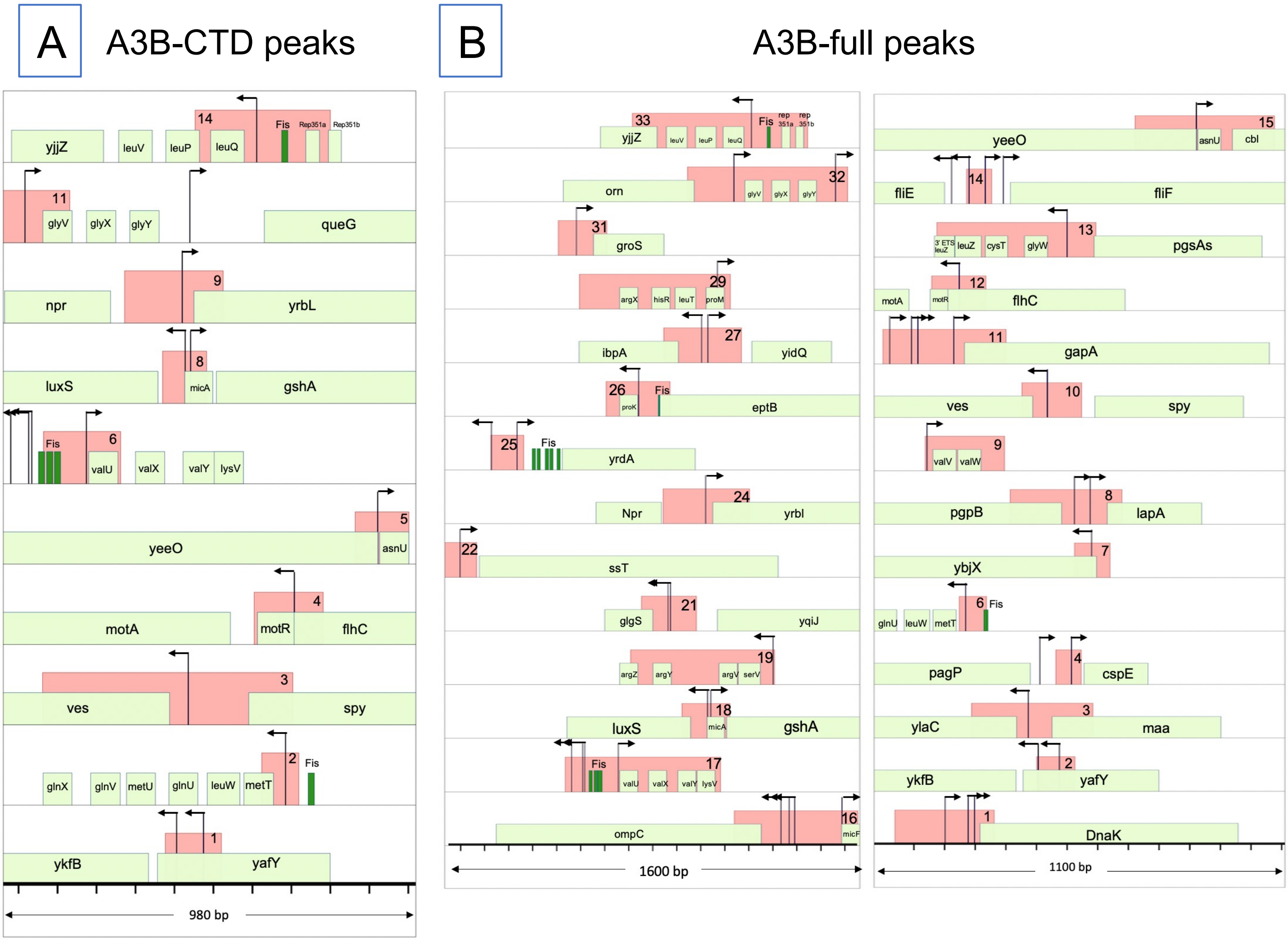

Salmon colored boxes are uracilation peaks created by A3B-CTD and A3B-full and *E. coli* genes with three letter name abbreviations are light green boxes. Experimentally determined transcription start sites (TSS) are vertical lines with left- or right-pointing arrows. The gene positions and TSS are according to EcoCyc database.

Supplementary Figure S4

Overlap between the Uracilation Peaks

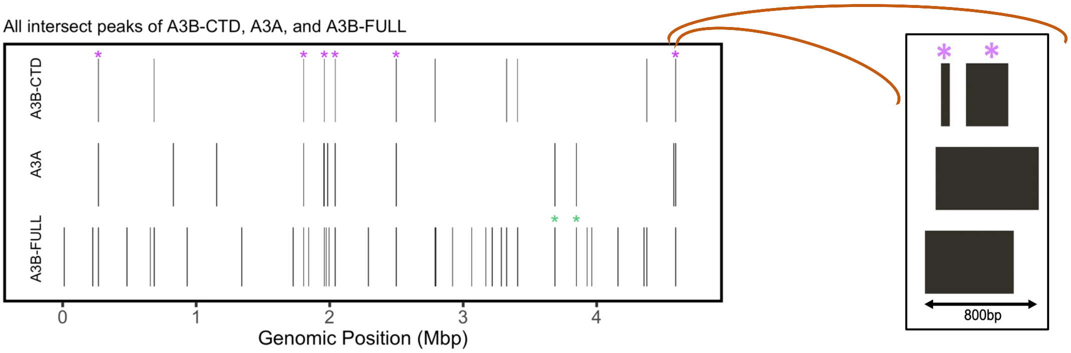

Supplementary Figure S5

Details of overlap between an overlapping uracilation peak for A3A, and A3B-CTD and A3B-full. A3B-full creates a uracilation peak between genome coordinates 4,605,937-4,606,667 that overlaps with a peak created by A3A. A3B-CTD generates two peaks (coordinates – 4,606,069-4,606,140 and 4,606,275-4,606,622) that overlap with the A3A and A3B-full generated peaks (Zoom-in figure on the right).

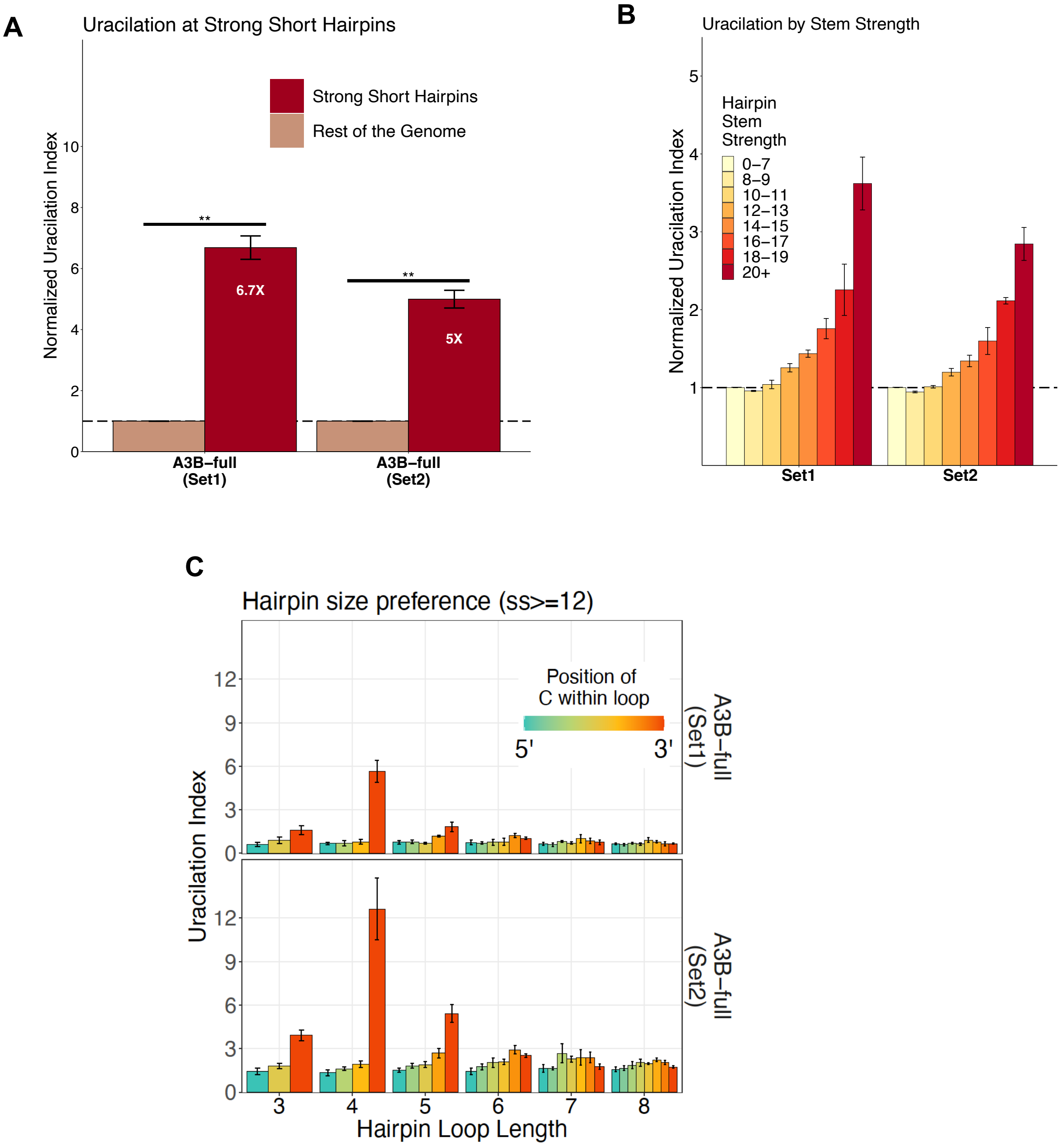

Supplementary Figure S6

Comparison of Two Sets of A3B-full UPD-seq data. UPD-seq was performed on biological triplicates of BH214 cultures expressing A3B-full at two different times. These are called Set 1 and Set 2. **A.** Preference of A3B-full for hairpin loops. **B.** Dependance of uracilation in hairpin loops on stem-strength. **C.** The preference of A3B-full for cytosines in different loop sizes and positions.

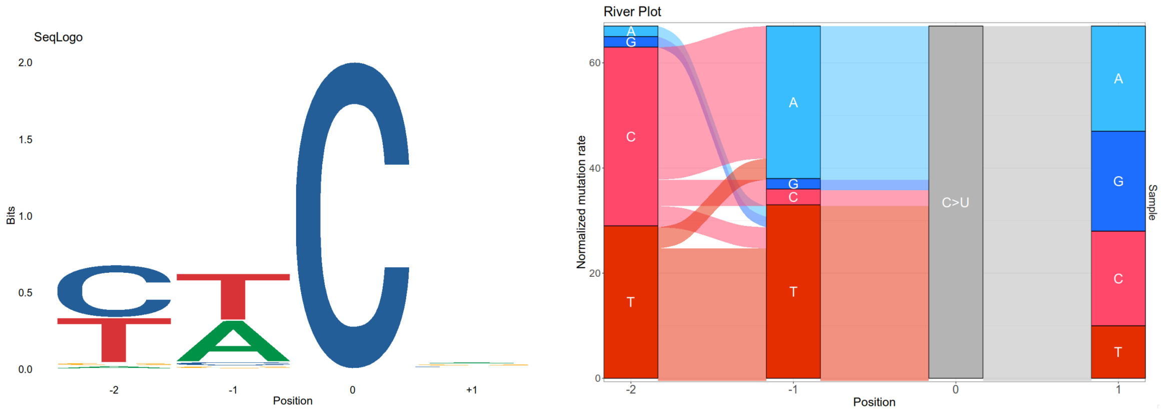

Supplementary Fig. S7

Comparison of SeqLogo and River plots. While sequence logo plots are useful for showing enrichment of mutations in particular sequence contexts, they lack the ability to convey any relational information with regards to the enriched bases. In our made-up example for a set of deamination events, the Seqlogo plot (Left panel) shows that A and T are enriched at -1 position of a targeted C, while C and T are enriched at the -2 position. Using a River plot to represent the same set of base changes (Right panel), we see that an A at the -1 position is more likely to be preceded by a C. Similarly, a T at the -1 position is more likely to be preceded by another T. When interpreting River plots, one should note the connectivity between different bases and the relative widths of different streams, as these indicate the prevalence of each mutation type and context. An inspection of the River plot, unlike the SeqLogo plot, allows us to conclude that TTC and CAC are preferred targets while TAC and CTC are not.

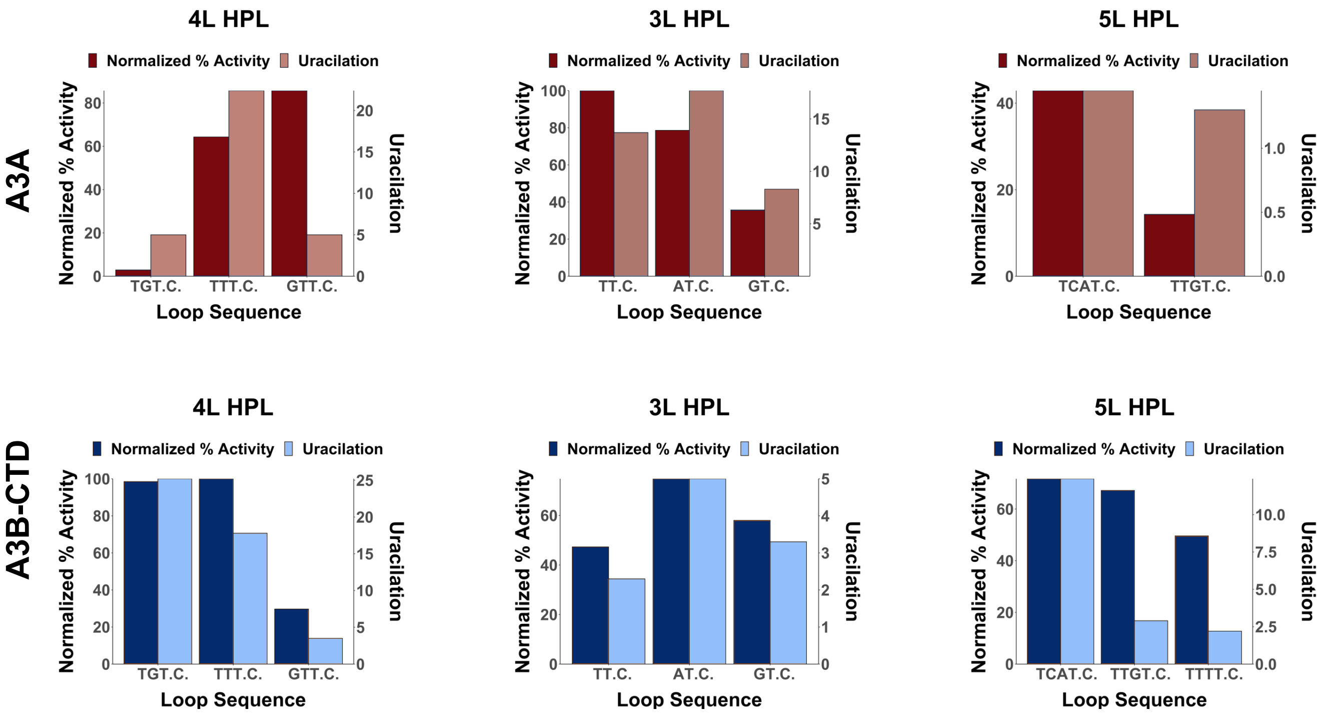

Supplementary Fig. S8

Biochemical activity of A3A and A3B-CTD for different hairpin loops. A3A and A3B-CTD activity normalized to the activity for the best substrate for each enzyme; TT.C. for A3A and TGT.C. for A3B-CTD

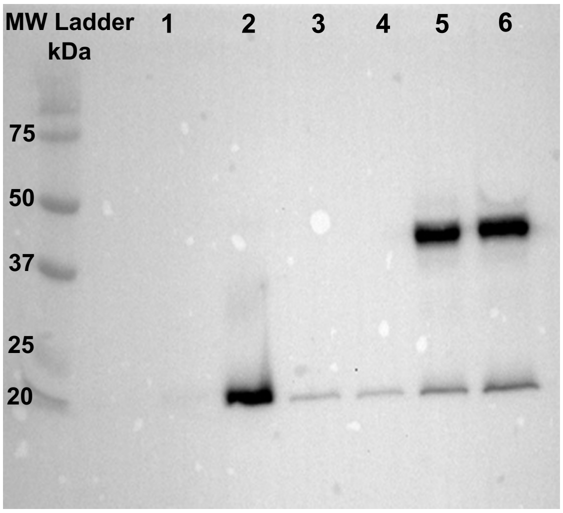

Lane	Sample	AHT concentration (µg/mL)	Quantified A3B or A3B-CTD (nmol)
1	Purified A3B-CTD (50 ng)	-	-
2	Purified A3B-CTD (150 ng)	-	-
3	Cell-free extract (A3B-CTD)	0.05	4.8
4	Cell-free extract (A3B-CTD)	0.1	3.7
5	Cell-free extract (A3B-Full)	0.05	17.1
6	Cell-free extract (A3B-Full)	0.1	24.9

Supplementary Fig. S9

Western blot analysis of expression of A3B-CTD and A3B-full in *E. coli*. Cell-free extracts and purified A3B-CTD were separated on an SDS-PAGE gel, transferred to a nylon membrane and interrogated using a rabbit anti-A3B monoclonal antibody (1:2000 dilution, NIH AIDS Reagent Program, catalog number 5210-87-13). The concentration of inducer, anhydrotetracycline (AHT) used, if any, and the source of protein loaded in each lane is noted in the Table on the right. The signal in lane 2 was used to calculate the amount of protein in lanes 3 through 6.

Supplementary Table S1
A3B-CTD

Sr. no	Uracilation peak* Start	Uracilation peak* End	Gene type	Gene	Targeted by A3B-full as well?	TSS
1	266130	266274	CDS	yafY	yes	266158
						266227
2	697103	697198	tRNA	metT	yes	697163
3	1824611	1825253	CDS	ves	yes	1824984
			CDS	spy	yes	
4	1977200	1977377	CDS	motR	yes	1977302
			CDS	flhC	yes	
5	2059788	2059900	CDS	yeeO	yes	2059845
			tRNA	asnU	yes	
6	2520815	2521013	tRNA	valU	yes	2520924
7	2521221	2521276	tRNA	valY	yes	no
			tRNA	lysV	yes	
8	2814745	2814858	CDS	micA	yes	2814802
						2814816
9	3348274	3348527	CDS	yrbl	yes	3348421
10	3428663	3428787	rRNA	rRNA	no	no
11	4392203	4392429	tRNA	glyV	yes	4392313
12	4392619	4392701	tRNA	glyY	yes	no
13	4606069	4606140	tRNA	leuV	yes	no
14	4606275	4606622	tRNA	leuP	yes	4606432
			tRNA	leuQ	yes	
			extragenic	rep351a	yes	
			extragenic	rep351b	yes	

*Uracilation peaks in the strain BH214 were detected using Normalized Differential Coverage (NDC) 2 software. The corresponding sequences in MG1655 were identified using the homology search engine within the EcoCyc Database and were annotated based on the information in EcoCyc {Keseler, 2021 #341}. CDS are protein coding genes. tRNA genes are shown in red.

Supplementary Table S1

A3B-full

Sr. no	Uracilation peak* Start	Uracilation peak* End	Gene type	Gene	Targeted by A3B-CTD as well?	TSS
1	11887	12209	CDS	dnaK	no	12048
						12123
						12144
2	266154	266279	CDS	yafY	yes	266158
						266227
3	479104	479500	CDS	ylaC	no	479288
			CDS	maa	no	
4	657200	657283	intergenic	metT	no	657250
5	696594	696789	tRNA	glnV	no	no
			tRNA	metU		
			tRNA	glnW		
6	697143	697232	intergenic		yes	697163
7	919048	919164	CDS	ybjX	no	919103
8	1339926	1340291	CDS	pgpB	no	1340135
			CDS	lapA	no	1340186
9	1746410	1746669	tRNA	valV	no	1746415
			tRNA	valW		
10	1824902	1825097	CDS	ves	yes	1824984
11	1862505	1862907	CDS	gapA	no	1862526
						1862597
						1862618
						1862735
12	1977213	1977390	srrna	motR	yes	1977302
			CDS	flhC	yes	
13	1991756	1992275	srrna	3' ETS leuZ	no	1992180
			tRNA	leuZ		
			tRNA	cysT		
			tRNA	glyW		
			CDS	pgsA		
14	2013086	2013168	intergenic		no	2013093
						2013146
15	2059646	2060101	tRNA	yeeO	yes	2059845
			tRNA	asnU	yes	
			CDS	cbl	no	
16	2312637	2313152	CDS	ompC	no	2312864
			CDS	micF	no	2312887
						2312830
						2313084
17	2520704	2521351	tRNA	valU	yes	2520730

			tRNA	valX	no	2520776
			tRNA	valY	yes	2520784
			tRNA	lysV		2520924
18	2814697	2814897	CDS	luxS	no	2814802
			CDS	micA	yes	2814816
			CDS	gshA	no	
19	2818105	2818704	tRNA	argZ	no	2818697
			tRNA	argY		
			tRNA	argV		
			tRNA	serV		
20	3086265	3086520	CDS	yqgC	no	no
			CDS	yqgD		
21	3191892	3192121	CDS	glgS	no	3192002
						3192012
22	3239796	intergenic			no	3239863
23	3308447	3308550	CDS	nlpl	no	no
24	3348245	3348605	CDS	yrbL	yes	3348421
25	3428939	3429076	intergenic		no	3428940
						3429047
26	3708560	3708826	tRNA	proK	no	3708694
			CDS	eptB	no	
27	3867361	3867685	CDS	ibpA	no	3867518
						3867544
28	3946944	3947047	tRNA	aspT	no	no
			tRNA	trpT		
29	3982425	3982836	tRNA	argX	no	3982781
			tRNA	hisR		
			tRNA	leuT		
			tRNA	proM		
30	4175423	4175956	tRNA	thrU	no	no
			tRNA	tyrU		
			tRNA	glyT		
			tRNA	thrT		
			CDS	tufB		
31	4370541	4370743	CDS	groS	no	4370616
32	4392121	4392788	CDS	orn	no	4392313
			tRNA	glyV	yes	4392736
			tRNA	glyX	no	
			tRNA	glyY	yes	
33	4605937	4606667	CDS	yjjZ	no	4606432
			tRNA	leuV	yes	
			tRNA	leuP		
			tRNA	leuQ		
			extragenic	rep351a		
			extragenic	rep351b		

*Uracilation peaks in the strain BH214 were detected using Normalized Differential Coverage (NDC2) software. The corresponding sequences in MG1655 were identified using the homology search engine within the EcoCyc Database and were annotated based on the information in EcoCyc {Keseler, 2021 #341}. CDS are protein coding genes. tRNA genes are shown in red.

Supplementary Table S2
Correlation between hairpin loops preferred by A3A, A3B-CTD and A3B-full

	A3A_1	A3A_2	A3A_3	A3A_4	A3B_CTD_1	A3B_CTD_2	A3B_CTD_3	A3B_full_1	A3B_full_2	A3B_full_3	A3B_full_4	A3B_full_5	A3B_full_6
A3A_1	1	0.5953623	0.7082655	0.6716858	0.3137248	0.352919	0.3514978	0.3758714	0.3720424	0.4058631	0.3286559	0.2515169	0.2824636
A3A_2	0.5953623	1	0.6530038	0.6346622	0.3137123	0.3768321	0.3530299	0.3956832	0.3852548	0.4258857	0.3401083	0.2551982	0.2664238
A3A_3	0.7082655	0.6530038	1	0.8205131	0.4201809	0.4589042	0.4324308	0.4404088	0.4493596	0.4515312	0.429623	0.3190364	0.3624017
A3A_4	0.6716858	0.6346622	0.8205131	1	0.3950985	0.4525015	0.4025707	0.4056686	0.3987843	0.4077256	0.4154228	0.3112035	0.3427507
A3B_CTD_1	0.3137248	0.3137123	0.4201809	0.3950985	1	0.7960863	0.7845507	0.7695222	0.7872811	0.777655	0.7200013	0.6556866	0.626292
A3B_CTD_2	0.352919	0.3768321	0.4589042	0.4525015	0.7960863	1	0.8076474	0.8035099	0.8186589	0.818538	0.7572724	0.690803	0.632426
A3B_CTD_3	0.3514978	0.3530299	0.4324308	0.4025707	0.7845507	0.8076474	1	0.7921193	0.8117791	0.8093987	0.7384234	0.6580035	0.6523893
A3B_full_1	0.3758714	0.3956832	0.4404088	0.4056686	0.7695222	0.8035099	0.7921193	1	0.8741952	0.8616117	0.741043	0.6560404	0.6103436
A3B_full_2	0.3720424	0.3852548	0.4493596	0.3987843	0.7872811	0.8186589	0.8117791	0.8741952	1	0.8800569	0.7516146	0.6897404	0.6129017
A3B_full_3	0.4058631	0.4258857	0.4515312	0.4077256	0.777655	0.818538	0.8093987	0.8616117	0.8800569	1	0.7420341	0.6692421	0.6137751
A3B_full_4	0.3286559	0.3401083	0.429623	0.4154228	0.7200013	0.7572724	0.7384234	0.741043	0.7516146	0.7420341	1	0.6445453	0.5945571
A3B_full_5	0.2515169	0.2551982	0.3190364	0.3112035	0.6556866	0.690803	0.6580035	0.6560404	0.6897404	0.6692421	0.6445453	1	0.5371814
A3B_full_6	0.2824636	0.2664238	0.3624017	0.3427507	0.626292	0.632426	0.6523893	0.6103436	0.6129017	0.6137751	0.5945571	0.5371814	1

	min	mean	max
Correlation values among A3A samples	0.5953623	0.6805821	0.8205131
Correlation values among A3B-CTD samples	0.7845507	0.7960948	0.8076474
Correlation values among A3B-full samples	0.5371814	0.6985922	0.8800569
Correlation values between A3A and A3B* samples	0.2515169	0.3733968	0.4589042

Table S3

The sequences of oligonucleotides used in this study

	Name	Sequence
1	Linear GTT.C.	5′-(6-FAM)-GCAAGCTGTTCAAAAAAATGA
2	TT.C.	5′-(6-FAM)-GCAAGCTTTCAGCTTGCTGA
3	GTT.C.	5′-(6-FAM)-GCAAGCTGTTCAGCTTGCTGA
4	AGTT.C.	5′-(6-FAM)-GCAAGCTAGTTCAGCTTGCTGA
5	TTT.C.	5′-(6-FAM)-GCAAGCTTTTCAGCTTGCTGA
6	10 nt oligo	5'-AGCTTGCTGA-(6-FAM)-3'
7	AT.C.	5′-(6-FAM)-GCAAGCTATCAGCTTGCTGA
8	GT.C.	5′-(6-FAM)-GCAAGCTGTCAGCTTGCTGA
9	TGT.C.	5′-(6-FAM)-GCAAGCTTGTCAGCTTGCTGA
10	TCAT.C.	5′-(6-FAM)-GCAAGCTTCATCAGCTTGCTGA
11	TTGT.C.	5′-(6-FAM)-GCAAGCTTTGTCAGCTTGCTGA
12	TTTT.C.	5′-(6-FAM)-GCAAGCTTTTTCAGCTTGCTGA

The stem sequence of hairpins is from NUP93[38]
