# Supplementary material for: Distinguishing preferences of human APOBEC3A and APOBEC3B for cytosines in hairpin loops, and reflection of these preferences in APOBEC-signature cancer genome mutations
Source: Nat Commun. 2024 Mar 18;15:2369. doi: 10.1038/s41467-024-46231-w (PMC10948833; doi:10.1038/s41467-024-46231-w)
Supplement: Supplementary file 3 — Description of Additional Supplementary Files [file 41467_2024_46231_MOESM3_ESM.pdf]

Description of Additional Supplementary Files

File Name: Supplementary Data 1

Description: This table lists all the UI (uracilation index) values for different 3 nt (nucleotide), 4 nt and 5 nt hairpin loops in the *Escherichia coli* genome when A3A, A3B-CTD or A3B-full was expressed in the cells. The UI values are defined in the main text. The table columns are loop length, position of cytosine in the loop (loop position), the expressed enzyme (sample), loop sequence (the cytosine that is changed to T is indicated by flanking dots- .C.), number of occurrences of a loop sequence in the genome, UI value and standard deviation (s.d.).
