# Supplementary material for: Distinguishing preferences of human APOBEC3A and APOBEC3B for cytosines in hairpin loops, and reflection of these preferences in APOBEC-signature cancer genome mutations
Source: Nat Commun. 2024 Mar 18;15:2369. doi: 10.1038/s41467-024-46231-w (PMC10948833; doi:10.1038/s41467-024-46231-w)
Supplement: Supplementary file 6 — Source data [file 41467_2024_46231_MOESM6_ESM.zip › Source data Files/Source Data Files A3B-CTD and A3A activity assays.docx]

**A3B-CTD activity assays**

**
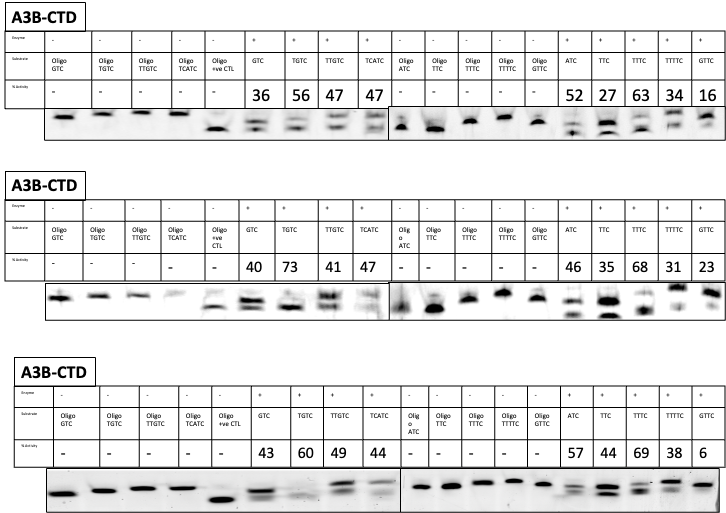
**The gel pictures below show all the gel pictures and the quantification of the product using ImageJ software. These numbers were used to calculate the correlation between A3A or A3B-CTD activity and UI values mentioned in the text.

**A3B-CTD**


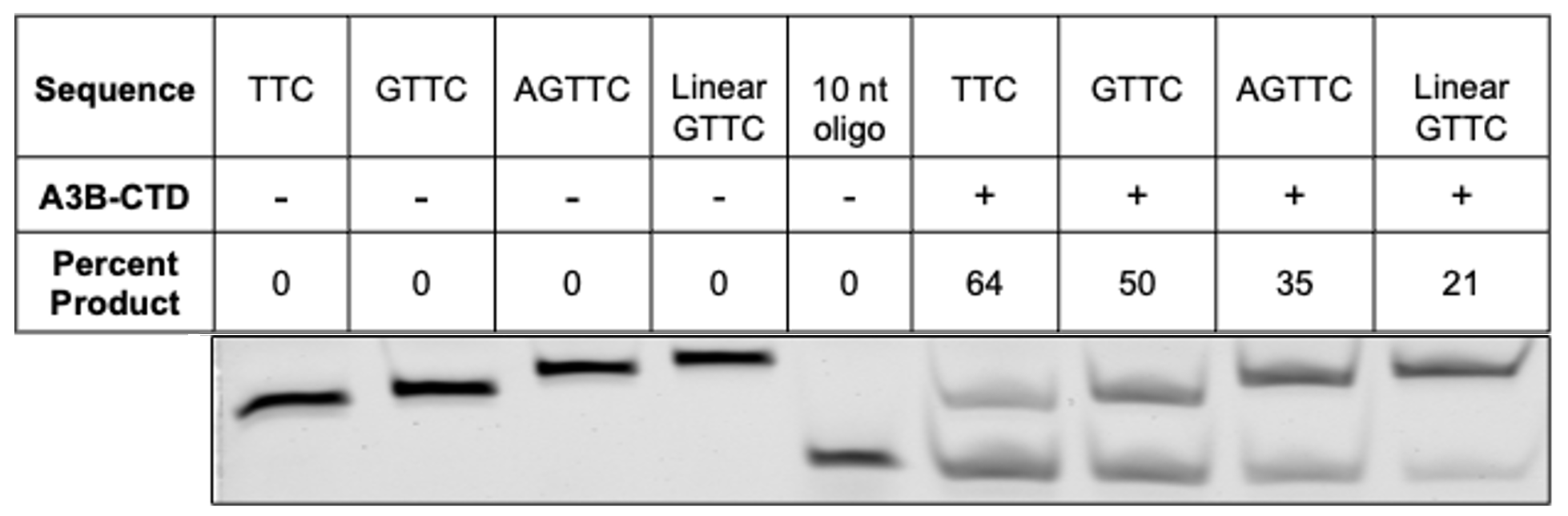


**A3A activity Assays**

**
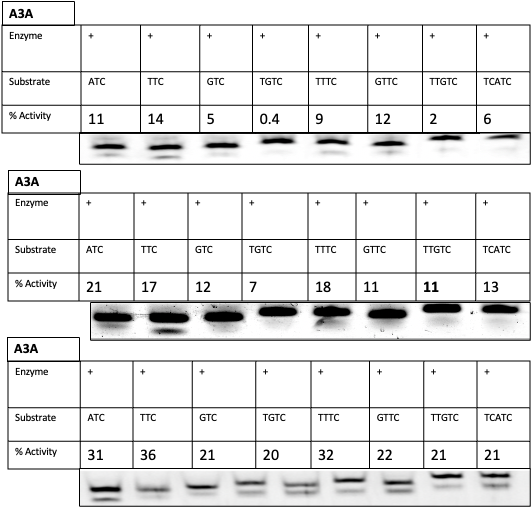
**
