# Supplementary material for: Distinguishing preferences of human APOBEC3A and APOBEC3B for cytosines in hairpin loops, and reflection of these preferences in APOBEC-signature cancer genome mutations
Source: Nat Commun. 2024 Mar 18;15:2369. doi: 10.1038/s41467-024-46231-w (PMC10948833; doi:10.1038/s41467-024-46231-w)

**Figure 4**

The figures 4B, 4C, 4D and 4E and based on gel-based activity assays. The original gel pictures used for Figs. 4B and 4C are shown below.

Figure 4B


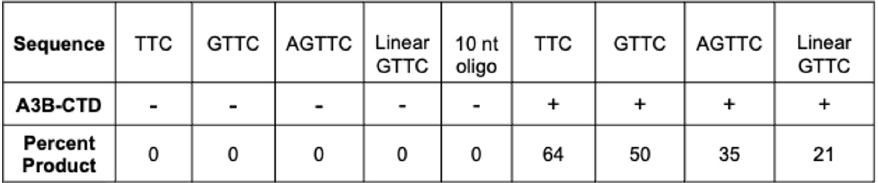


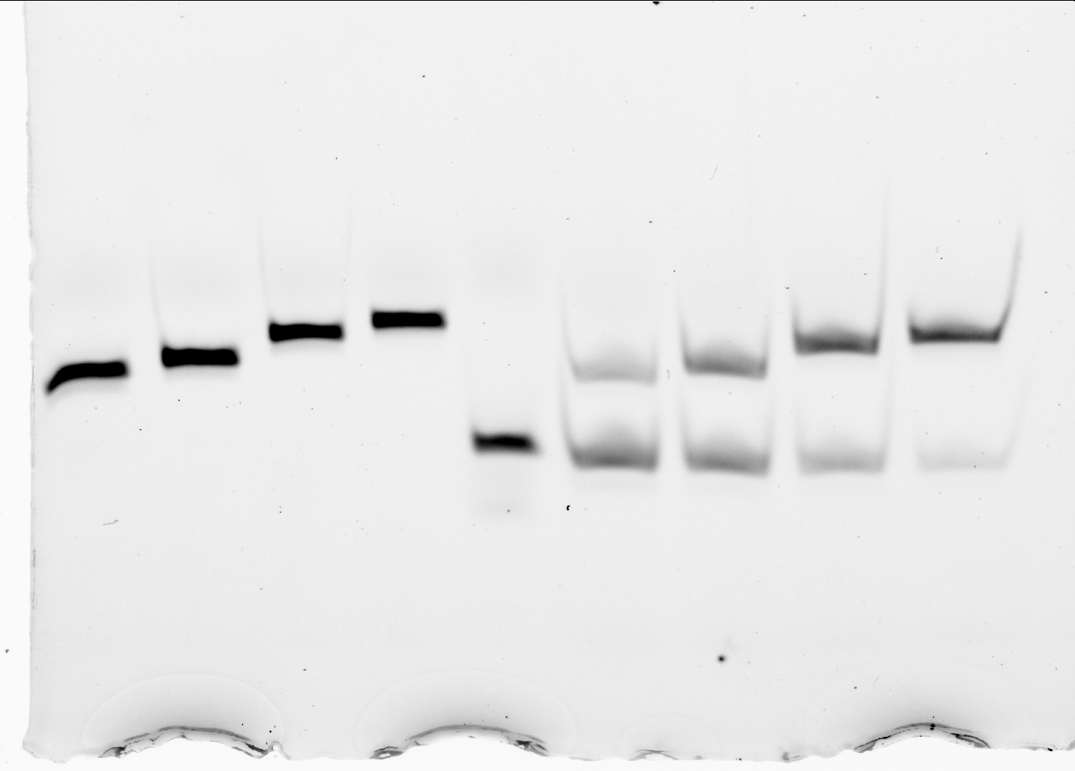


Figure 4C


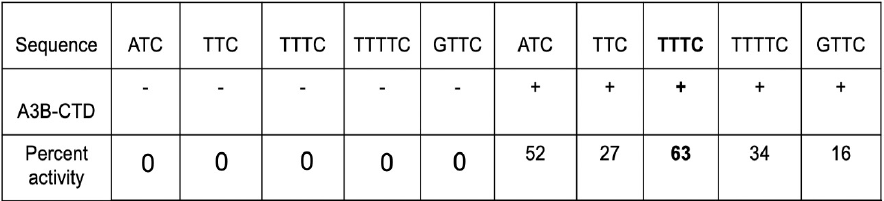


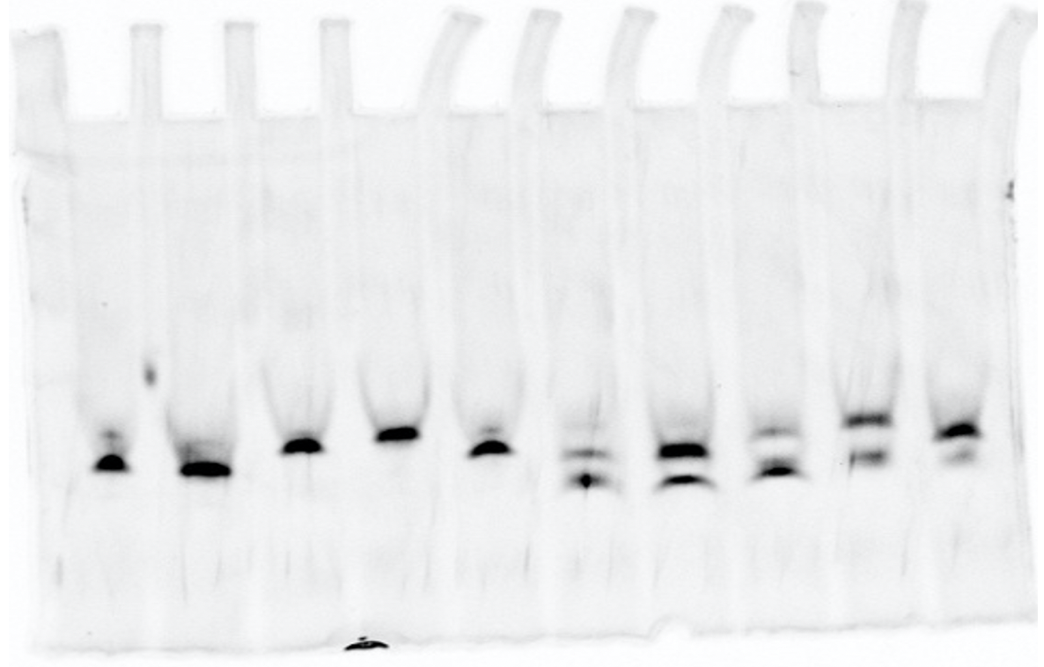

Supplement: Supplementary file 6 — Source data [file 41467_2024_46231_MOESM6_ESM.zip › Source data Files/Source Data File Figure 4 Original images.docx]
