# Supplementary material for: Distinguishing preferences of human APOBEC3A and APOBEC3B for cytosines in hairpin loops, and reflection of these preferences in APOBEC-signature cancer genome mutations
Source: Nat Commun. 2024 Mar 18;15:2369. doi: 10.1038/s41467-024-46231-w (PMC10948833; doi:10.1038/s41467-024-46231-w)
Supplement: Supplementary file 6 — Source data [file 41467_2024_46231_MOESM6_ESM.zip › Source data Files/Source Data File Supplementary Figure S9.docx]

**Supplementary Figure S9**

Original scan of the Western blot


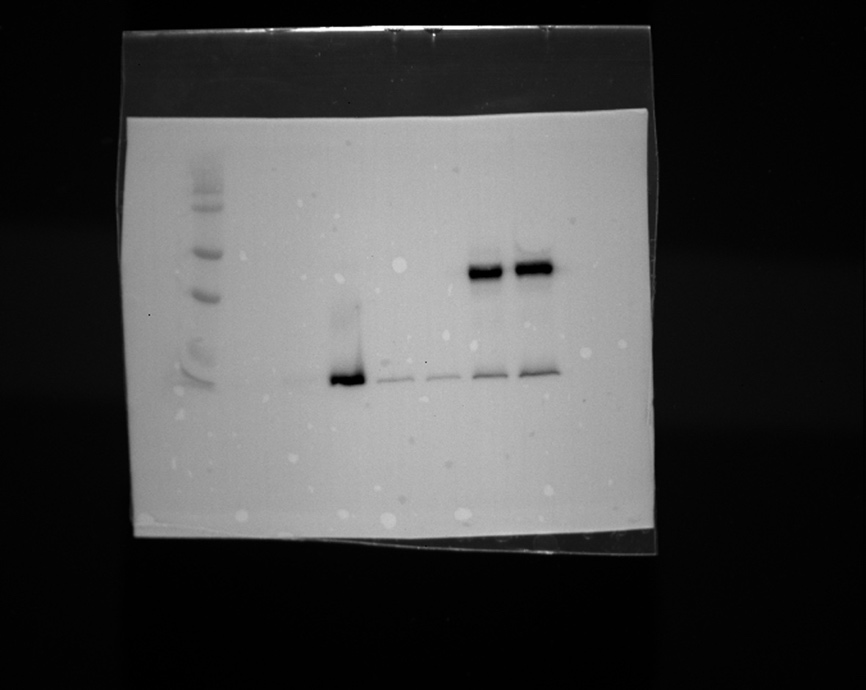


The antibody used- Cell Signaling Technology Catalog #5210-87-13. Rabbit mAb #81001 against APOBEC3A/B/G.
