# Supplementary figures and images for: Distinguishing preferences of human APOBEC3A and APOBEC3B for cytosines in hairpin loops, and reflection of these preferences in APOBEC-signature cancer genome mutations
Source: Nat Commun. 2024 Mar 18;15:2369. doi: 10.1038/s41467-024-46231-w (PMC10948833; doi:10.1038/s41467-024-46231-w)

**ImageJ Measurements and analysis**

**Figure 4B**


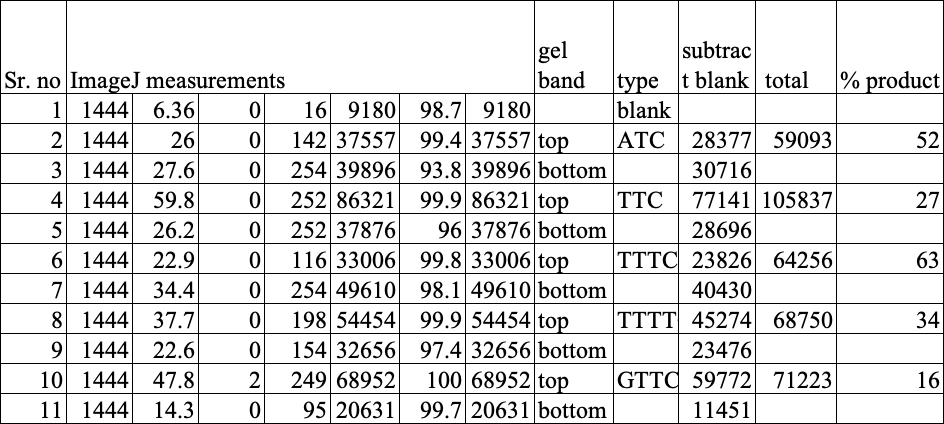


**Figure 4C**

**
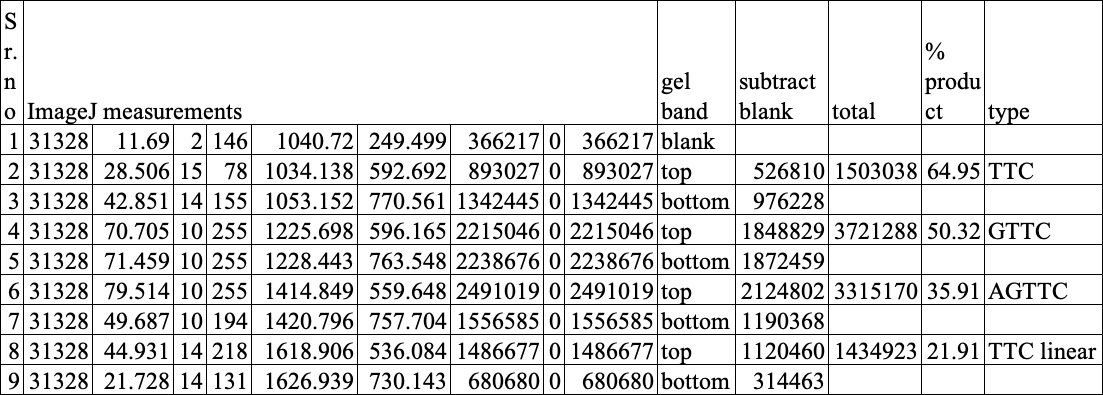
**

Supplement: Supplementary file 6 — Source data [file 41467_2024_46231_MOESM6_ESM.zip › Source data Files/Source Data File ImageJ Measurements and analysis Figures 4B and 4C.docx]
